# Consideration for Others

**Published:** 1920-11

**Authors:** C. N. Johnson


### CONSIDERATION FOR OTHERS

BY C. N. JOHNSON, D.D.S.

When I am in the washroom of a Pullman sleeper I can usually tell the men who are gentlemen and those who are not. There is something which stamps the real gentleman at once when he comes in contact with his fellowmen, and it is not so much elegance of diction, taste or richness of dress, exclusiveness of manner, or any of those attributes which are accounted paramount in the make-up of a gentleman—it is not so much these as it is that innate consideration for the rights and privileges of others which is the real foundation of all ethical relationship one with the other.

And in no place is this more conspicuously shown than among fellow-passengers in a sleeping-car. They are thrown so closely together in the limited space of a washroom that the amenities of life are more intimate and pronounced than elsewhere. And a real gentleman recognizes this and acts accordingly. He may, or may not, say a word to his fellow-passengers, but he goes unobtrusively about the affairs of his toilet without causing unnecessary inconvenience to others in the washroom. He does not spread his articles of apparel over the room and take up space out of all proportion to his rights. He shows some consideration for those about him.

And there is one little act above all others which seems to me most significant as stamping a man a gentleman or otherwise. It is this: after washing and splashing up the bowl, if he takes a towel and wipes it clean and dry for the next passenger he is at once raised in my estimation. Think of it! What right have you or I to make our fellow-passengers clean up after us? And this is precisely what many travelers are doing every day—some of them unconsciously being rude and imposing on others, while some are indifferent to the amenities, and care not a whit how much they inconvenience their fellowmen. If their injustice were ever called to their attention they would at once claim that it is the duty of the porter to clean up after the passengers, and some would arrogantly inform you that they were not traveling for the purpose of doing a porter's work. All of which is wrong—wrong in principle and wrong in practical application. In the first place, the porter could not well wipe the bowl after every passenger, and in the next place he is usually out in the car, very properly making up the berths. And anyhow, what right have you or I to go through the world having people wait on us hand and foot at every turn? By what circumstance of birth or position was it ordained that we should lord it over our fellowmen, and loll back helplessly all the while waiting to be served?

I am not foolish enough to affirm all men can go through the world on a perfect equality. Some have the mental and physical endowment for one kind of service, and some for another. There are men and women who lack the mental initiative to direct the affairs of the world, or to direct affairs of any kind. Some one must go ahead and lead them. They are fitted to do only what they are told to do, and their physical endowment is usually very much superior to their mentality. The fact that they must do the physical labor rather than the mental is apparent to every one; in other words, they are fitted for servants and for nothing else. Happy is the man who realizes his peculiar fitness for certain things, and who goes cheerfully to his allotted task and does it as well as he knows how.

But this does not give to others who are better endowed mentally the right to brutally lord it over the underling and impose on him such menial tasks, or in such lordly fashion, as to stunt his mentality still farther, and force him lower in the scale of self-respect. We should constantly aim to build up character and respect rather than to tear it down.

The lack of consideration which some people show for their fellowman is a disgrace to our civilization. It matters not that one individual may have more money than another—it matters not in the least. So many times has it been stated, and so often has it been demonstrated, that the possession of money does not make a gentleman of a man.

I liked very much the contention of the late Count Tolstoi, who claimed years ago that every boy and girl, no matter what their station in life, should be brought up to wait on themselves. That is, if they took a bath they should clean the tub after them and not leave it for some one else to do; if they misplaced any article, it should be put back in its proper place—in other words, that they should hold themselves responsible for correcting any disorder which they might create in their daily duties. How much better this than to bring up a child to the theory that he is superior to others, and that it is his inalienable right to be waited upon.

I recall a boy from a supposedly fine family who got out of my chair one day and threw the napkin carelessly on the floor—for some one to pick up. I was sorry for that boy; I knew he had a lesson to learn. And he got the lesson. He was of draft age when the great war broke out, was taken to France, and served in the ambulance division for many weary, nerve-racking months. When he came home he was a changed boy, a lovable boy. Now he is most solicitous for others, most considerate of their point of view, most charming as an individual. He learned his lesson, and is much happier in every way than before. The war was not all in vain.

Every child should be trained to a sense of his individual responsibility. It should be forced upon him that when he constantly does things which add to the burden and impose hardship upon others he is committing a deadly sin, which will react upon him in a myriad ways. A wrong point of view is a calamity to any individual, and the worst of all points of view is the one which instills into any man's mind the theory that he can go through life without consideration for his fellowmen.

